# Adherence to AHA Guidelines When Adapted for Augmented Reality Glasses for Assisted Pediatric Cardiopulmonary Resuscitation: A Randomized Controlled Trial

**DOI:** 10.2196/jmir.7379

**Published:** 2017-05-29

**Authors:** Johan N Siebert, Frederic Ehrler, Alain Gervaix, Kevin Haddad, Laurence Lacroix, Philippe Schrurs, Ayhan Sahin, Christian Lovis, Sergio Manzano

**Affiliations:** ^1^ Geneva Children’s Hospital Department of Pediatric Emergency Medicine University Hospitals of Geneva Geneva Switzerland; ^2^ Division of Medical Information Sciences Department of Radiology and Medical Informatics University Hospitals of Geneva Geneva Switzerland; ^3^ Geneva Medical Center University Hospitals of Geneva Geneva Switzerland

**Keywords:** resuscitation, emergency medicine, pediatrics, biomedical technologies, equipment and supplies, eyeglasses

## Abstract

**Background:**

The American Heart Association (AHA) guidelines for cardiopulmonary resuscitation (CPR) are nowadays recognized as the world’s most authoritative resuscitation guidelines. Adherence to these guidelines optimizes the management of critically ill patients and increases their chances of survival after cardiac arrest. Despite their availability, suboptimal quality of CPR is still common. Currently, the median hospital survival rate after pediatric in-hospital cardiac arrest is 36%, whereas it falls below 10% for out-of-hospital cardiac arrest. Among emerging information technologies and devices able to support caregivers during resuscitation and increase adherence to AHA guidelines, augmented reality (AR) glasses have not yet been assessed. In order to assess their potential, we adapted AHA Pediatric Advanced Life Support (PALS) guidelines for AR glasses.

**Objective:**

The study aimed to determine whether adapting AHA guidelines for AR glasses increased adherence by reducing deviation and time to initiation of critical life-saving maneuvers during pediatric CPR when compared with the use of PALS pocket reference cards.

**Methods:**

We conducted a randomized controlled trial with two parallel groups of voluntary pediatric residents, comparing AR glasses to PALS pocket reference cards during a simulation-based pediatric cardiac arrest scenario—pulseless ventricular tachycardia (pVT). The primary outcome was the elapsed time in seconds in each allocation group, from onset of pVT to the first defibrillation attempt. Secondary outcomes were time elapsed to (1) initiation of chest compression, (2) subsequent defibrillation attempts, and (3) administration of drugs, as well as the time intervals between defibrillation attempts and drug doses, shock doses, and number of shocks. All these outcomes were assessed for deviation from AHA guidelines.

**Results:**

Twenty residents were randomized into 2 groups. Time to first defibrillation attempt (mean: 146 s) and adherence to AHA guidelines in terms of time to other critical resuscitation endpoints and drug dose delivery were not improved using AR glasses. However, errors and deviations were significantly reduced in terms of defibrillation doses when compared with the use of the PALS pocket reference cards. In a total of 40 defibrillation attempts, residents not wearing AR glasses used wrong doses in 65% (26/40) of cases, including 21 shock overdoses >100 J, for a cumulative defibrillation dose of 18.7 Joules per kg. These errors were reduced by 53% (21/40, *P*<.001) and cumulative defibrillation dose by 37% (5.14/14, *P*=.001) with AR glasses.

**Conclusions:**

AR glasses did not decrease time to first defibrillation attempt and other critical resuscitation endpoints when compared with PALS pocket cards. However, they improved adherence and performance among residents in terms of administering the defibrillation doses set by AHA.

## Introduction

Clinical practice guidelines aim to improve quality of care, reduce variation of practice, and provide evidence-based health care [1]. The American Heart Association (AHA) guidelines for cardiopulmonary resuscitation (CPR) are nowadays recognized as the world’s most authoritative resuscitation guidelines [2,3]. They are evidence-based, synthesized by experts, and include a large number of algorithms intended to provide step-by-step processes to various life-threatening emergency situations in a systematic fashion. These algorithms are also summarized on pocket reference cards in order to be used as quick reference tools that emergency physicians may have access to during resuscitations. However, despite their availability, suboptimal quality of resuscitation is still common for both adult and pediatric patients [4]. Immediate (level 1) triage represents 175,000 patient visits every year in US pediatric emergency departments (PED) [5]. Among them, 5800 to 10,000 cases are due to in-hospital cardiac arrest (IHCA) [6,7], and 6700 to 15,000 cases to out-of-hospital cardiac arrest (OHCA) [8-10], including 6000 related to non-traumatic causes [11]. Quality CPR with adherence to AHA resuscitation guidelines optimizes the management of critically ill patients and increases their chances of survival [12,13], whereas deviation is associated with decreased likelihood of survival from cardiac arrest (CA) [14]. Currently, the median hospital survival rate from pediatric IHCA is 36% [4], whereas it is below 10% for OHCA [15,16].

As a result, the scientific community has proposed new resuscitation strategies relying on information technologies and devices aiming at improving and ensuring adherence to AHA guidelines [17-20]. Among possible emerging information technologies that could support caregivers, augmented reality (AR) glasses have recently gained a great deal of interest within the scientific community. AR glasses are wearable and connected devices that display interactive images to the visual field of users by overlaying visual information without significantly disturbing the ordinary vision. They feature some functionalities similar to those offered by smartphones and tablets by running self-contained mobile apps. Despite recent communications and studies related to the use of these glasses in various medical fields [21-26], their contribution to resuscitation in emergency medicine has not yet been investigated. Their potential to wirelessly display and interact in real time conditions with data generated by the actions performed in a specific environment could be of great interest for assisting caregivers in resuscitation rooms, while freeing users’ hands and allowing them to “see the scene through the screen”. Augmented reality might bring useful information to caregivers’ attention without disturbing the care process, by allowing constant access to the resuscitation scene. In a previous article, we have described the adaptation of AHA Pediatric Advanced Life Support (PALS) algorithms for AR glasses [27]. This study aimed at investigating whether this adaptation for AR glasses would increase adherence to AHA guidelines by reducing deviation and time to initiation of critical life-saving maneuvers during pediatric CPR when compared with the use of PALS pocket reference cards, in a simulated model.

## Methods

### Study Design

We conducted a prospective, randomized controlled trial with 2 parallel groups of voluntary pediatric residents comparing time to first defibrillation attempt while using AR glasses (Google Glass, allocation group A) or AHA PALS conventional pocket reference cards (allocation group B) during a standardized simulation-based pediatric CA scenario (Multimedia Appendix 1). No changes were made to the AR glasses or the intervention during the study.

#### Selection of Participants

Any physician from the whole pediatric department actively training for a pediatric specialty (residents) was eligible. Residents with eye disorders were not included in the study. Shift-working residents were randomly recruited on the day of the study from a random alphabetical list. Written informed consent was obtained from all the participants before their voluntary involvement. Study participants were not involved in the study design, choice of outcome measures, or the execution of the study. No participants were asked for advice on interpretation or the writing of results. The results of the study were offered to the study participants after the completion of the study.

#### Setting and Resuscitation Scenario

The study was conducted in a pediatric emergency department (PED) of a tertiary hospital with approximately 28,000 visits per year. We created a standardized simulation scenario on a high-fidelity manikin (Laerdal SimJunior). The resuscitation team was composed of the resident participating in the study and 3 nurses to assist with resuscitation through drugs preparation, chest compressions, and bag-valve-mask ventilation, according to the resident’s instructions. A certified technician (KH), who was not a member of the resuscitation team, operated the simulator. Except for participating residents, members of the resuscitation team remained unchanged across all scenarios and were the investigators in the study.

**Figure 1:**
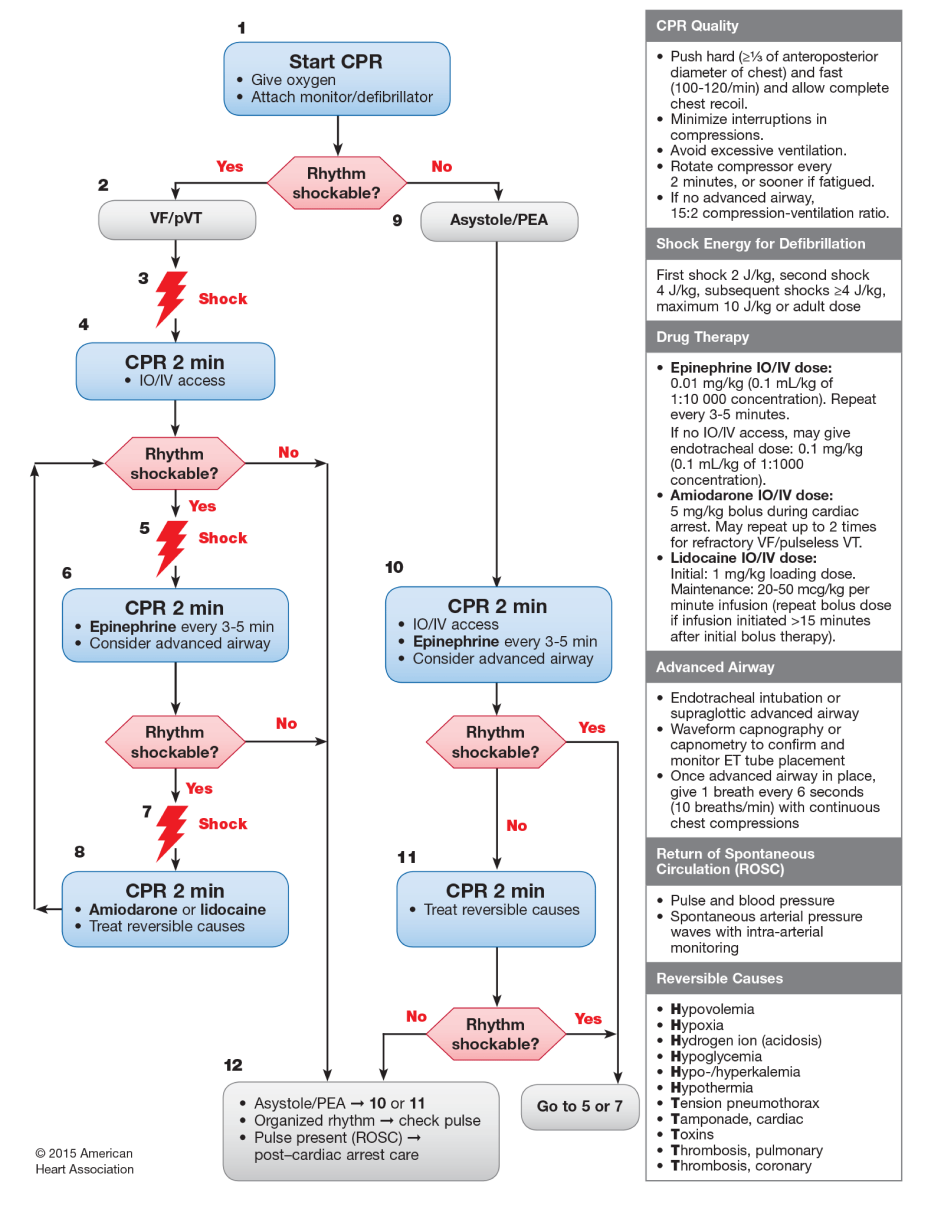
American Heart Association’s pediatric cardiac arrest algorithm—2015 update.

**Figure 2:**
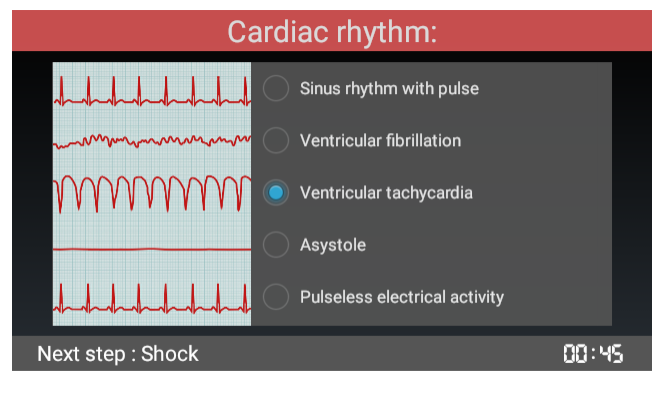
Screenshot of a resuscitation step from the American Heart Association’s Pediatric Advanced Life Support pulseless ventricular tachycardia algorithm as adapted in augmented reality glasses.

On the day of participation, residents were asked about their demographics. After random allocation, each participant allocated to the AR glasses group received a standardized 15-minute qualifying training session to familiarize them uniformly with the AR glasses. Then, all the participants were asked to perform a 15-minute highly realistic CPR scenario (Multimedia Appendices 2 and 3). Unlike adults, CA in children without prior cardiac disease is mainly due to asystole (40%) and pulseless electrical activity (24%) [28]. Ventricular fibrillation and pulseless ventricular tachycardia (pVT), namely shockable rhythms, are identified in 27% of pediatric IHCA [29]. We decided to study the pVT algorithm because, in our opinion, it offers a greater opportunity to assess the multiple-steps resuscitative skills set by AHA. The scenario was therefore standardized to strictly follow the 2015 AHA pediatric pVT algorithm (Figure 1) [30] and provided on the same manikin. It was conducted in situ in the pediatric resuscitation room of the PED to increase realism. No interactions occurred between participants and investigators. When entering the room, a short clinical statement to recognize the life-threatening condition of the patient, including his weight and age, was given to the resident. The resident was then asked to start the timed scenario and had to recognize by himself or herself the previously settled cardiac rhythm (pVT) with the AR glasses (allocation group A) or the PALS conventional pocket reference card (group B). All participants in allocation group B were required to have their PALS pocket card in their hands throughout the entire scenario. Whether they referred to it or not was left to them, as in real life. The scenario ran invariably until the manikin was defibrillated at the 4th shock and showed a subsequent return of spontaneous circulation (ROSC). In order to be consistent with the 2015 AHA pediatric CA algorithm [30] and to standardize our scenario, defibrillation doses of 2 Joules per kg for the first attempt, and 4 Joules per kg for subsequent 2nd, 3rd, and 4th attempts were expected (Figure 1).

#### Intervention

As previously described [27], the numerous steps of AHA PALS algorithms were wisely split into “cards.” Each card transposed to the AR glasses paralleled the informational content of a resuscitation step from the original algorithm. However, the informational content was “augmented,” thanks to the interactivity and display capability of the device. The AR algorithm thus obtained was set up in a manner similar to the PALS pocket references regarding the progression and sequence of actions along the original algorithm’s sequences. For instance, the complete pulseless ventricular tachycardia (pVT) algorithm was adapted on 42 cards designed to be as concise as possible without hindering proper progression along the algorithm. The cards were tailored to the small size of the Google glass screen, following a user-centered and ergonomic-driven approach. Each card was structured on 4 zones: (1) a color-coded title allowing direct identification of each step in progress, (2) an image on the left helping with decision-making (such as distinctive illustration of cardiac rhythms), (3) a menu choice on the right helping to progress in the resuscitation steps, and (4) a footer to preview the next step (Figure 2). Interaction was also defined with end users. Tactile commands of the glasses were favored over voice commands due to the inability of AR glasses to distinguish between vocal orders in the noisy environment of a resuscitation room. Swiping up or down allowed navigating inside the card. Selection was done by a click and actions cancelled by swiping back. Each cycle of chest compression–ventilation was timed, thanks to a countdown clock displayed on the screen. Treatment and defibrillation doses (Philips HeartStart MRx Biphasic Defibrillator) were automatically calculated on patients’ weight or age.

#### Outcome Measures

The elapsed time in seconds in each allocation group from onset of pulseless shockable rhythm to the first defibrillation attempt was selected as the primary outcome, as it is the most important determinant of survival after CA [31]. Secondary outcomes were time elapsed to initiation of chest compression, time to subsequent defibrillation attempts, time to administration of epinephrine and amiodarone, and time interval (in seconds) between defibrillation attempts. AHA recommends 5 cycles of chest compression (about 2 minutes) between defibrillation attempts. The amount of time spent by participants to perform chest compressions by cycles of chest compression was defined as the hands-on time. It was measured in seconds with a chronometer. Drug doses, shock doses, and number of shocks were also assessed. All these outcomes were assessed for deviation from AHA guidelines. At the end of the scenario, a questionnaire using a 10-point Likert scale was submitted to the participant to measure the overall stress perceived during the scenario.

#### Methods of Measurement and Data Collection

All the actions (ie, the primary and secondary outcomes) performed by the resident during the scenario were automatically recorded and stored by the responsive simulator detectors and by several video cameras. The videos were embedded in a dedicated simulation software, allowing accurate assessment of timing and sequencing of actions. To avoid assessment bias, 2 evaluators then independently reviewed these video recordings. In case of disagreement, a third independent evaluator helped reach a consensus. Data were manually retrieved and entered into a Microsoft Excel spreadsheet (version 2011). Unaccomplished actions were left blank and not assigned any corresponding time. Only residents were assessed and their privacy preserved. Only the investigators of the study had access to the data. The statistical software GraphPad Prism version 6.0h (GraphPad Software, Inc) was used for all data analyses.

#### Sample Size

The primary objective of the study was to detect a difference in time to the first defibrillation attempt. The sample size was calculated to detect a 30-second decrease in time to first defibrillation between 2 independent groups with a power of 80% and a 2-sided risk alpha of .05. A previous study has shown a mean time to first defibrillation of 92 seconds [32] with a standard deviation (SD) of 23 seconds. Assuming a similar SD in each group in our study, 10 patients per group were required.

#### Randomization and Blinding

We randomly assigned residents in a 1:1 ratio with a Web-based software [33]. Blinding to the purpose of the study during recruitment was maintained to minimize preparation bias. Participants were unblinded after randomization. Allocation concealment was ensured with the same Web-based software [33] and was not released until the residents started the scenario.

#### Statistical Analysis

##### Primary Outcome

We first evaluated the time elapsed between onset of pVT and first defibrillation attempt. The Shapiro-Wilks test was used for normality analysis of the parameters. Means and standard deviations (SDs) with 95% CI were reported. Non-normally distributed variables were analyzed using the Mann-Whitney test. Frequencies were reported as percentages. *T* tests were used to compare independent groups. No paired data were compared. Kaplan–Meier curves for time elapsed between onset of pVT and 1) initiation of chest compression, and 2) first defibrillation, were estimated and compared using the log-rank (Mantel-Cox) test for bivariate survival analysis.

##### Secondary Outcomes

We evaluated the time elapsed between onset of pVT to subsequent defibrillation attempts and drug delivery. As most of the continuous variables were also normally distributed, means and SDs with 95% CI were reported. Non-normally distributed variables were analyzed using a Mann-Whitney test. Frequencies were reported as percentages. *T* tests were used to compare independent groups. No paired data were compared. Errors in cycles of chest compression-ventilation were measured as the deviation in percent from the experimental time spent in seconds compared with the 2-minute duration recommended by AHA guidelines. Wrong defibrillation or drug doses were measured as the deviation in percent from the amount of energy delivered in Joules or drug doses in milliliters compared with AHA recommendations. Wrong defibrillation mode was also measured. Absolute deviations were also analyzed. The mean (SD) difference in deviation obtained with each method was reported with 95% CI. A *t* test for unpaired data was used to compare interventions. Mean differences were reported by randomized group. We also determined if prior certification as a PALS provider before the study, prior resuscitations as a provider in real-life, or post-graduation years (PGY) had a significant impact on the above outcomes. Mean and SD were determined for stress scores of individuals in the questionnaire and reported with descriptive statistics. A *P* value less than .05 was considered significant.

#### Ethics and Informed Consent

The study was approved by the institutional ethics committee. According to the ICMJE, a registration number was not required for our trial, as the purpose of this study was to examine the effect of the intervention on the providers. Written informed consent was obtained from all participants before their voluntary involvement. The study was conducted in accordance with the principles of the Declaration of Helsinki, the standards of Good Clinical Practice, and Swiss regulatory requirements.

**Figure 3:**
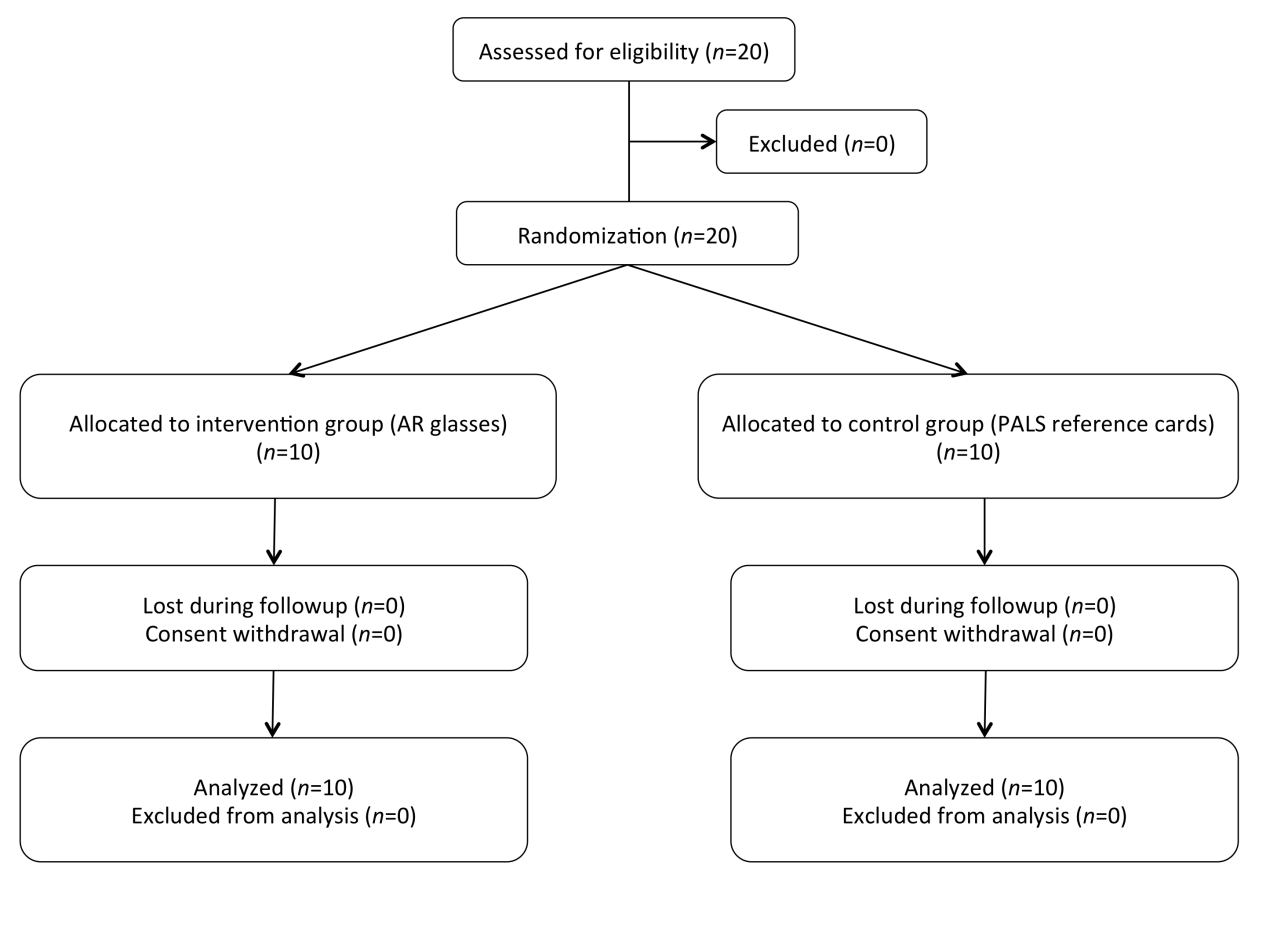
CONSORT flowchart of augmented reality glasses trial.

## Results

### Study Participants

In March 2016, 20 pediatric residents participated and completed the study with no dropout (Figure 3). The demographic results are summarized in Table 1.

### Time to Resuscitation Critical Endpoints

**Figure 4:**
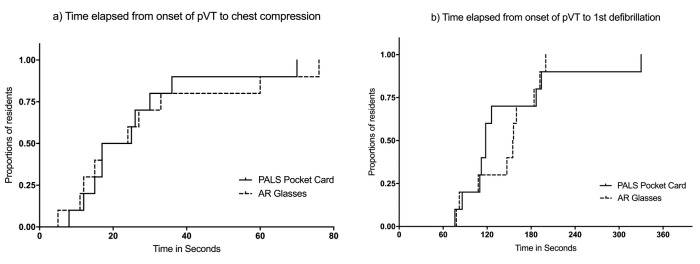
Kaplan–Meier curves of proportion of pediatric residents using augmented reality glasses or conventional Pediatric Advanced Life Support pocket cards who a) initiated chest compression, or b) delivered first defibrillation shock during a simulated pulseless ventricular tachycardiapVT scenario (Log-rank test statistic, P=.81 and P=.99).

The first defibrillation was delivered within 180 seconds by 70% of the residents in both groups (Figure 4). Mean times to resuscitation critical endpoints are summarized in Table 2. None of them were significantly different between allocation groups A and B. All participants (100%) correctly recognized the pVT rhythm on the monitor. In both allocation groups, 90% of the residents initiated chest compressions within 60 seconds from the onset of pVT, and half of them before 20 seconds. There were no statistically significant differences in hands-on time spent by cycles of chest compression between both groups. Due to a lack of some defibrillation attempts, 1 interval in allocation group A and 4 in group B were not measurable.

At the time of the study, 6 participants (60%) in allocation group A and 7 participants (70%) in allocation group B were PALS-certified providers. Eleven participants out of 20 (55%) were residents with more than 1 year of PGY. With regard to all outcomes measured, we observed in both allocation groups that PALS-certified residents or those with PGY>1 tended to defibrillate and deliver drugs more quickly than non-PALS residents or those with ≤1 PGY (Multimedia Appendix 4). We observed no difference with previous resuscitation experience (data not shown).

**Table 1:** Participants’ demographics and clinical characteristics.

Demographics and clinical characteristics	Randomization Arm	
	AR^a^ Glasses (n=10)	PALS^b^ pocket cards (n=10)
Age in years, mean (SD)	27.9 (2.6)	29.2 (2.6)
Sex (female), n (%)	9 (90)	6 (60)
Years of residency, mean (SD)	2.4 (1.7)	2.4 (1.7)
Number of residents having been enrolled in > 5 resuscitations in the past, n (%)	4 (40)	3 (30)
Number of PALS providers among residents, n (%)	6 (60)	7 (70)
Number of BLS^c^ providers among residents, n (%)	10 (100)	10 (100)
Level of self-confidence in following AHA guidelines (on a scale of 1 to 5), mean (SD)	1.8 (1.2)	2.4 (0.8)

^a^AR: Augmented Reality.

^b^PALS: Pediatric Advanced Life Support.

^c^BLS: Basic Life Support.

**Table 2:** Mean time to resuscitation critical endpoints.

Outcomes	AR^a^ Glasses Mean (SD, 95% CI)		PALS^b^ pocket cards Mean (SD, 95% CI)	Time difference^c^	*P* value
Time to initiation of CPR^d^	28.0 (22.9, 11.6-44.4)		25.6 (17.8, 12.9-38.3)	2.4	.80
Time to 1st defibrillation attempt	146.2 (43.5, 115.1-177.3)		145.7 (75.1, 92.0-199.4)	0.5	.99
Time to 2nd defibrillation attempt	264.0 (73.9, 211.1-316.9)		263.0 (74.2, 210.0-316.0)	1.0	.98
Time to epinephrine	317.3 (62.6, 265.0-369.5)		295.8 (97.7, 220.7-370.9)	21.5	.59
Time to 3rd defibrillation attempt	396.6 (93.6, 329.7-463.5)		389.0 (80.0, 314.8-462.9)	7.7	.86
Time to amiodarone		450.1 (53.6, 408.9-491.3)	492.7 (106.5, 416.5-568.9)	42.6	.28
Time to 4th defibrillation attempt		542.8 (83.3, 478.7-606.8)	526.8 (93.4, 455.0-598.6)	16.0	.71

^a^AR: Augmented Reality.

^b^PALS: Pediatric Advanced Life Support.

^c^Time difference represents absolute time difference between PALS pocket cards and AR Glasses.

^d^CPR: cardiopulmonary resuscitation.

**Table 3:** Errors and deviations from the American Heart Association’s pulseless ventricular tachycardia algorithm.

Critical Resuscitation Endpoints (AHA pVT^a^ algorithm)	AHA^b^ recommended doses (Joules per kg), (mL per kg)	Randomization Arm				
		AR^c^ Glasses Mean (SD, 95% CI)		% deviation (*P* value)	PALS^d^ ref. cards Mean (SD, 95% CI)	% deviation (*P* value)^i^
1st defibrillation attempt	2.00	2.12 (0.71, 1.61-2.63)		6 (.60)	3.46 (2.23, 1.87-5.05)	73 (.068)
2nd defibrillation attempt	4.00	3.40 (0.97, 2.71-4.09)		15 (.081)	4.52 (1.53, 3.42-5.62)	13 (.31)
Epinephrine 1:10,000	0.1	0.1 (0, 0.1-0.1)		0 (1.00)	0.1 (0, 0.1-0.1)	0 (1.00)
3rd defibrillation attempt	4.00	4.00 (0, 4.00-4.00)		0 (1.00)	5.00 (1.51, 3.74-6.26)	25 (.10)
Amiodarone	0.1	0.1 (0, 0.1-0.1)		0 (1.00)	0.1 (0, 0.1-0.1)	0 (1.00)
4th defibrillation attempt	4.00	4.00 (0, 4.00-4.00)		0 (1.00)	5.68 (1.18, 4.84-6.52)	42 (.0014)
Cumulative defibrillation dose	14.0	13.52 (0.97, 12.32-14.72)		3.4 (.10)	18.66 (2.03, 13.87-23.45)^e^	33.3 (.025)
Correct AHA sequence		60%			40%^f^	
Correct number of shocks		80%			70%	
Shock overdoses (>100 J)		1/40 opportunities			21/40 opportunities^g^	
Total number of errors		5/40 opportunities			26/40 opportunities^h^	
Defibrillation errors at 1st attempt, n (details)		2 (40% lower, 200% higher)			5 (0.6 Joules per kg to 6 Joules per kg)	
Defibrillation errors at 2nd attempt, n (details)		3 (2 Joules per kg instead of 4 Joules per kg)			5 (1.2 Joules per kg to 6 Joules per kg)	
Defibrillation errors at 3rd attempt, n (details)		0			8 (2 Joules per kg to 6 Joules per kg)	
Defibrillation errors at 4th attempt, n (details)		0			8 (4.8 Joules per kg to 8 Joules per kg)	

^a^pVT: Pulseless ventricular tachycardia.

^b^AHA: American Heart Association.

^c^AR: Augmented Reality.

^d^PALS: Pediatric Advanced Life Support.

^e^Difference between AR glasses and PALS reference cards groups: *P*=.0010.

^f^Difference between AR glasses and PALS reference cards groups: *P*=.66.

^g^Difference between AR glasses and PALS reference cards groups: *P*<.001.

^h^Difference between AR glasses and PALS reference cards groups: *P*<.001.

^i^% deviation denotes percentage deviation from AHA recommended dose.

### Errors and Deviations From AHA pVT Algorithm

Errors and deviations from the AHA pVT algorithm are summarized in Table 3. Out of 40 opportunities, 5 errors in defibrillation doses (12.5%) were committed during the whole scenario in allocation group A. This compares to 26 errors in defibrillation doses (65%) during the whole scenario in allocation group B (*P*<.001). Out of 10, one resident (10%) in allocation group B wrongly used a synchronized shock, 2/10 (20%) never delivered a third defibrillation, and 2/10 (20%) stopped the compressions after the 1st or 2nd defibrillation attempts.

The entire pVT algorithm was followed correctly in a stepwise fashion until ROSC by 60% of residents in allocation group A and 40% in allocation group B (*P*=.66).

### Questionnaire About Perceived Stress and Satisfaction

The questionnaire was completed and returned by 100% of the participants. Participants in allocation group A and B rated the overall perceived stress to be 6.2 (95% CI 4.7-7.7) and 7.0 (95% CI 5.7-8.3), respectively on the Likert scale (*P*=.38). The usability, acceptance, and perception of the AR glasses were a major concern in our study and were assessed using a 17-item Unified Theory of Acceptance and Use of Technology (UTAUT) questionnaire [34]. The results will be published in a separate upcoming study.

## Discussion

### Principal Findings

To our knowledge, this is the first randomized controlled trial to investigate the benefit of a wearable technology to improve pediatric residents’ performance and adherence with regard to AHA resuscitation guidelines. Using AR glasses, we found that time to first defibrillation attempt, time to other critical resuscitation endpoints, and drug dose delivery were not improved in terms of adherence to AHA guidelines. However, errors and deviations from the pVT guideline in terms of defibrillation doses and cumulative defibrillation doses were significantly reduced when compared with the use of the PALS pocket reference cards.

During resuscitation, time is a decisive success criterion. During the first 15 minutes, survival and favorable neurological outcome decrease linearly by 2.1% and 1.2% per minute, respectively [35]. Delays in initiating CPR have a detrimental effect on patient outcome regardless of the quality of resuscitation [36]. AHA therefore recommends pulseless patients of any age to receive immediate CPR without delay starting with chest compressions followed by a defibrillation within 180 seconds of a shockable rhythm. However, management, procedural skills, and adherence to these guidelines have been shown to fade after a few months of initial training [37-39]. With a critical patient’s condition and stress, physicians do not always have enough time to apply these guidelines and are prone to deviate from them [40]. PALS pocket cards are intended to resolve this problem by delivering fast and accurate summarized resuscitative knowledge and skills to providers. Nevertheless, Hunt et al have observed that despite availability of these recommendations, 66% of pediatric residents failed to start compressions within 60 seconds from the onset of a simulated pVT, 33% never started compressions, only 54% successfully defibrillated within 180 seconds, and 7% never discharged the defibrillator [40]. Similar results were obtained by Labrosse et al during a simulated pulseless shockable arrest scenario, where 25% of pediatric residents failed to start compressions and 4% never defibrillated a patient [41]. A more recent study among first-year pediatric residents showed a median time for initiation of CPR of 50 seconds and to first defibrillation of 282 seconds [42]. Pediatric residents performed better in our study with delays closer to AHA recommendations with a mean time to initiate chest compressions of 25 to 28 seconds and to first defibrillation of 146 seconds. In both allocation groups, 90% of residents started compressions within 60 seconds from the onset of pVT, and 70% defibrillated within 180 seconds. However, there were no advantages for residents to wear the AR glasses as they performed similarly with or without them regarding delay to critical resuscitation endpoints, whether they were PALS-certified or not. Despite an ergonomic-driven approach to adapt AHA resuscitation algorithms in AR glasses and a prior 15-minute training session for their use, our system failed to improve resuscitation efficiency in terms of time to major endpoints. An explanation might be that reducing further time to defibrillations and drug delivery was not achievable by residents training in emergency medicine. Indeed, our results in accordance with those from Hunt et al [40] showed a trend toward improvement over PGY of training and PALS certification in the mean time to all defibrillation attempts. It would be interesting in further studies to assess this assumption with certified emergency physicians.

Current AHA resuscitation guidelines emphasize 2 minutes of chest compressions between defibrillations attempts as optimal care for persistent pVT or VF in children [30,43]. In this study, residents performed similarly on average, with or without AR glasses and close to AHA recommendations.

Prompt defibrillation is crucial for termination of VF or pVT to achieve ROSC [43]. The AHA 2015 guidelines recommend treating pVT or VF in children with an initial dose of 2 J/kg [30]. For subsequent shocks, a dose of 4 J/kg is recommended, though higher energy levels may be considered up to adult dose, if not exceeding 10 J/kg (Figure 1). In this trial, residents using the PALS pocket cards were more prone to deviate from defibrillation doses than those using the AR glasses. On average, the shocks they provided were delivered with defibrillation doses 13% to 73% above AHA recommendations. “High” defibrillation doses concerned mostly the initial shock, with doses reaching up to 6 J/kg in 40% of cases when delivered by residents not wearing the glasses. In an observational study of 285 pediatric IHCA, a higher initial shock dose of more than 2 J/kg was not associated with superior termination of pVT or VF or improved survival rates [44]. In addition, children who were defibrillated with higher initial shock doses in the >3-5 J/kg range were significantly less likely to have termination of pVT or VF with ROSC or to survive the event. In our study, the final cumulative defibrillation dose delivered by the residents in the PALS pocket cards group was on average 33% significantly higher than the AHA expected value. This deviation was 10 times greater than that seen with residents wearing the AR glasses. In particular, 50% of residents using the PALS pocket cards used wrong energy doses whether it was for first or second shock delivery. In a total of 40 defibrillation attempts, they used wrong doses in 65% of cases for a cumulative defibrillation dose of 18.7 J/Kg. These errors were reduced by 53% and cumulative dose by 37% by using the AR glasses, suggesting a limited but worthwhile benefit of their use in simulated resuscitation. It would be interesting in further studies to determine whether this would translate into fewer errors in shock doses in real life.

Finally, in terms of drug dose concentrations, both groups in this study accurately administered epinephrine and amiodarone. The entire pVT algorithm was followed correctly until ROSC in a stepwise fashion by 60% of residents wearing AR glasses, compared with 40% of residents with the PALS pocket cards.

### Limitations

This study has some limitations. First, it was conducted in a simulation-based resuscitation scenario. This choice was related to the ethical and organizational difficulties of conducting studies in real-life critical conditions. However, several studies have demonstrated the benefit of simulation as an investigative research methodology to answer research questions that otherwise could not be answered during CPR [45]. High-fidelity simulation is recognized as an essential tool to study resuscitation skills or technologies. Till date, none of the results obtained from simulation-based CPR studies disagreed with those obtained from studies in real life, confirming our study design choice. Realism was achieved, reflected by the stress levels experienced by the participants. They quoted the simulation as highly stressful when compared with real CPR situations.

Second, one might raise questions of our choice to choose Google Glass as the best AR glasses to display AHA guidelines. Indeed, despite its remarked 2012 commercial unveiling, Google Glass never reached its public audience and was discontinued in January 2015. However, there is a growing interest in recent literature toward its use in specialized medical fields [21,22,25,46,47]. We hypothesize that other AR glasses would not drastically change the results that we found with Google Glass, as their small size remains a major limitation to their use in displaying CPR algorithms. Further studies would be valuable to assess this assumption.

### Conclusions

Taken together, our results support the interpretation that residents are not accurately following AHA recommendations during pediatric resuscitation, whether they are PALS certified or not. A wearable technology such as AR glasses might partially fill this gap and benefit patients by improving adherence and performance of residents to meet resuscitation requirements set by AHA, especially regarding delivery of defibrillation doses. In this sense, AR glasses appear as an interesting tool for emergency medicine and future studies are required to further examine this new paradigm.

## Appendix

Multimedia Appendix 1 CONSORT-EHEALTH V1.6 checklist.

Multimedia Appendix 2 Use of AR glasses to guide resuscitation.

Multimedia Appendix 3 Following the pVT pathway of the AHA algorithm when adapted for augmented reality glasses during a simulation-based pediatric cardiac arrest scenario.

Multimedia Appendix 4 The table details the mean time from onset of pVT to the study main endpoints in both PALS and non-PALS residents.

## Abbreviations

AHA: American Heart Association
AR: Augmented Reality
CA: Cardiac Arrest
CPR: Cardiopulmonary Resuscitation
IHCA: In-Hospital Cardiac Arrest
OHCA: Out-of-Hospital Cardiac Arrest
PALS: Pediatric Advanced Life Support
PED: Pediatric Emergency Department
PGY: Post-Graduation Years
pVT: pulseless ventricular tachycardia
ROSC: Return Of Spontaneous Circulation
VF: Ventricular Fibrillation
