# Supplementary figures and images for: Adherence to AHA Guidelines When Adapted for Augmented Reality Glasses for Assisted Pediatric Cardiopulmonary Resuscitation: A Randomized Controlled Trial
Source: J Med Internet Res. 2017 May 29;19(5):e183. doi: 10.2196/jmir.7379 (PMC5468544; doi:10.2196/jmir.7379)

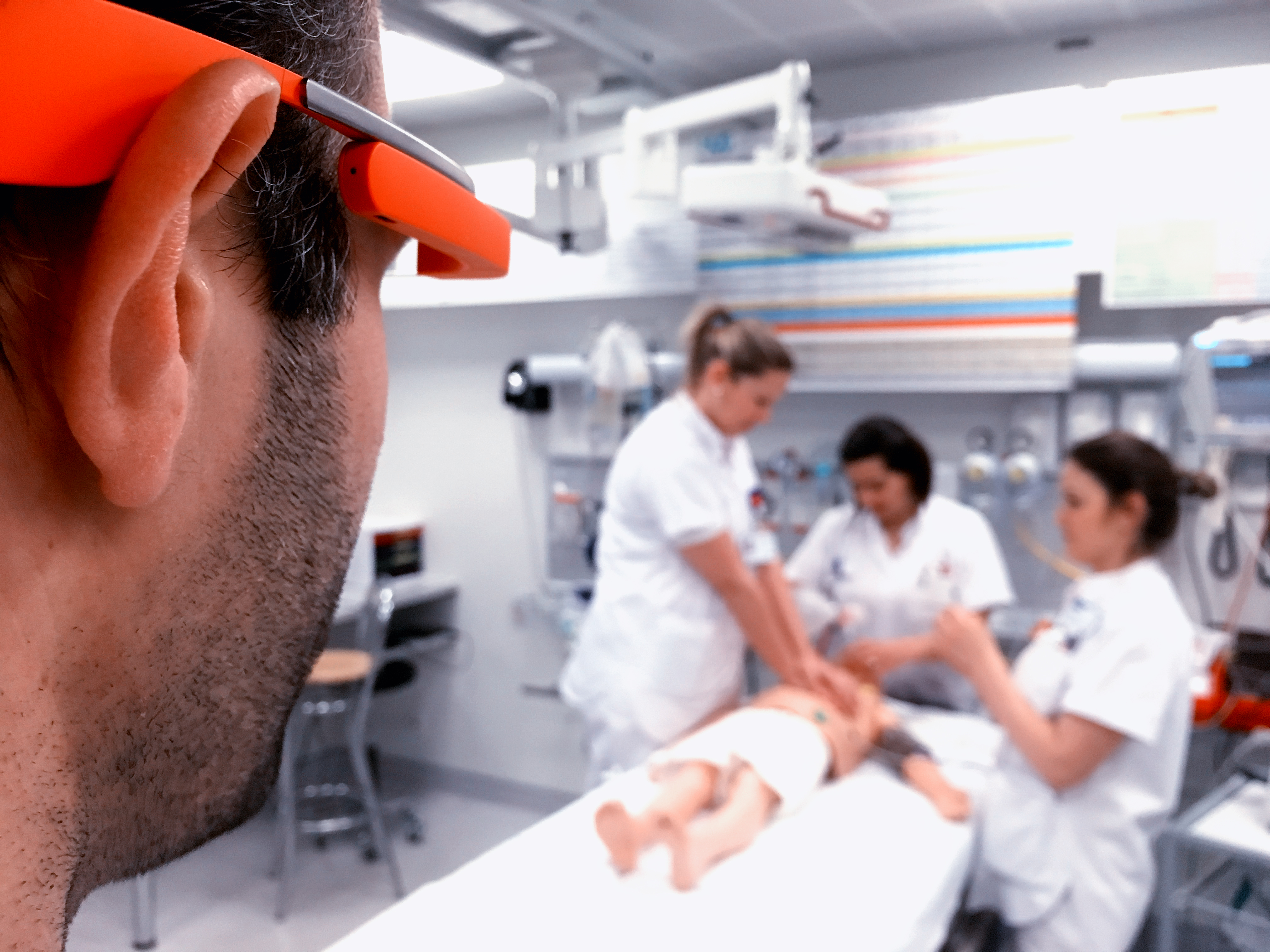

Supplement: Multimedia Appendix 2 [file jmir_v19i5e183_app2.jpg]

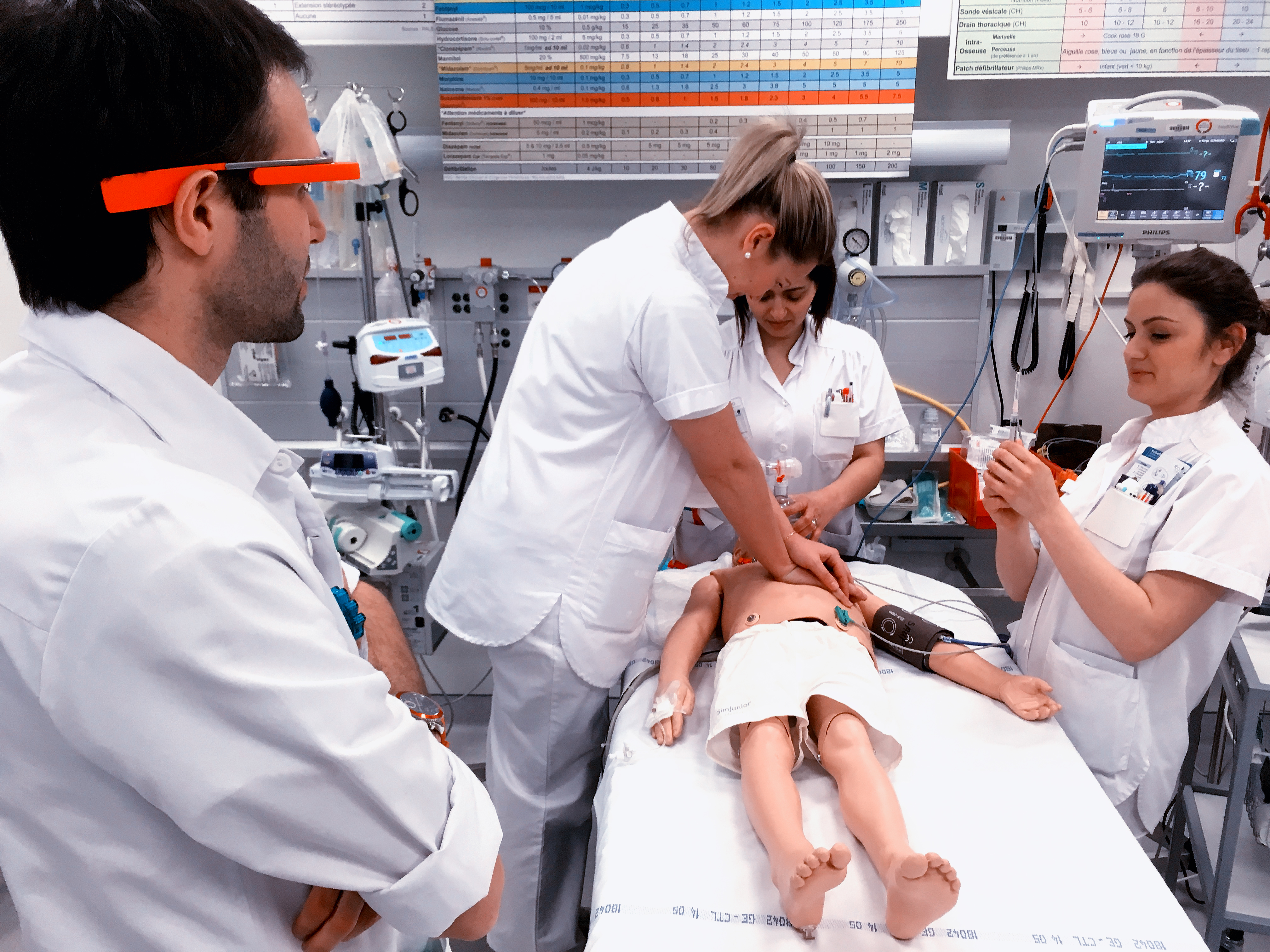

Supplement: Multimedia Appendix 3 [file jmir_v19i5e183_app3.jpg]

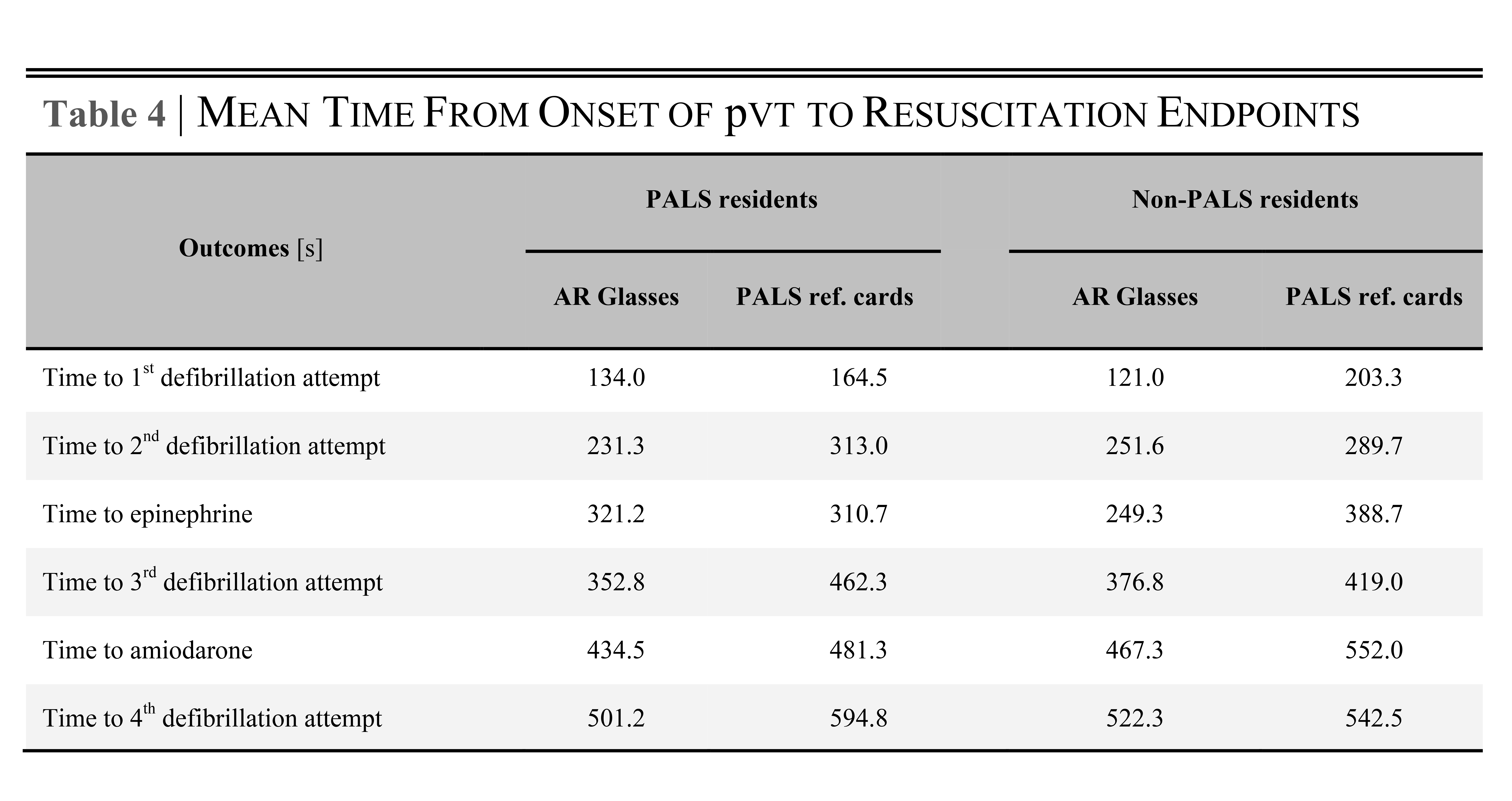

Supplement: Multimedia Appendix 4 [file jmir_v19i5e183_app4.png]
